# Crystal structures of 2′-benzoyl-1′-(4-methyl­phenyl)-1,1′,2,2′,5′,6′,7′,7a′-octa­hydro­spiro­[indole-3,3′-pyrrolizin]-2-one and 2′-(4-bromo­benzoyl)-1′-(2-chloro­phen­yl)-1,1′,2,2′,5′,6′,7′,7a′-octa­hydro­spiro­[indole-3,3′-pyrrolizin]-2-one

**DOI:** 10.1107/S2056989016016741

**Published:** 2016-10-25

**Authors:** M. Chandrarekha, N. Srinivasan, P. Kottala Vijaya, A. Siva, R. V. Krishnakumar

**Affiliations:** a Department of Physics, Thiagarajar College, Madurai 625 009, Tamil Nadu, India; b School of Chemistry, Madurai Kamaraj University, Madurai 625 021, Tamil Nadu, India

**Keywords:** crystal structure, indoline-3,3′-pyrrolizin derivatives, hydrogen bonding

## Abstract

The two title compounds, C_28_H_26_N_2_O_2_, (I), and C_27_H_22_BrClN_2_O_2_, (II), differ in their substituents, *viz*.4-methyl­phenyl and benzoyl rings in (I) replaced by 2-chloro­phenyl and 4- bromo­benzoyl, respectively, in (II). A significant difference between the two mol­ecules is found in the deviation of the benzoyl O atom from the least-squares plane of the ring to which it is attached [0.593 (4) and 0.131 (3) Å, respectively], a fact which may be attributed to the different participation of the benzoyl O atoms as acceptors in their inter­molecular C—H⋯O inter­actions. The chemical modifications in (I) and (II) do not seem to affect the type nor strength of the inter­molecular N—H⋯N and C—H⋯O hydrogen bonds responsible for the two crystal structures, such that the aggregation of mol­ecules appears similar in spite of the mol­ecular changes.

## Abstract 2

The chemical modifications in terms of changes in substituents in the title compounds have not affected the type nor strength of two defining inter­molecular inter­actions present in both crystal structures.

## Chemical context   

Pyrrolizine, a bicyclic ring system containing two fused pyrrole rings, is present in many herbs (Hoang *et al.*, 2015 ▸) and displays a variety of biological activities such as anti­convulsant (Abbas *et al.*, 2011 ▸), anti­arrhythmic (Miyano *et al.*, 1983 ▸), anti­viral (Kadushkin *et al.*, 1990 ▸), anti­bacterial (Sing *et al.*, 2002 ▸) *etc*. Indole, a pharmacologically significant nucleus, is known for anti-inflammatory (Misra *et al.*, 1996 ▸), anti­bacterial (Dandia *et al.*, 1993 ▸) and anti­viral (Giampieri *et al.*, 2009 ▸) activities.
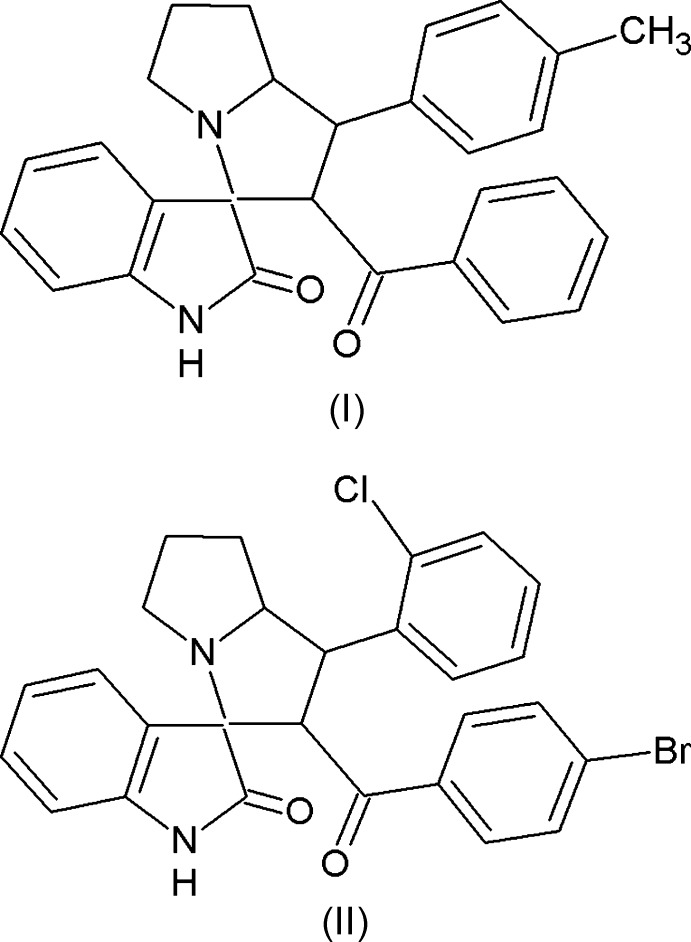


The title compounds (I) and (II) are ­spiro compounds in which the pyrrolizine and indole rings are spiro-fused, in addition to having respective benzo­yl/methyl­phenyl and bromo­benzo­yl/chloro­phenyl substitutions. In the present work, the mol­ecular and crystal structures of (I) and (II) are presented, and the differences in their mol­ecular conformation and inter­molecular inter­actions is discussed.

## Structural commentary   

**Figure 1:**
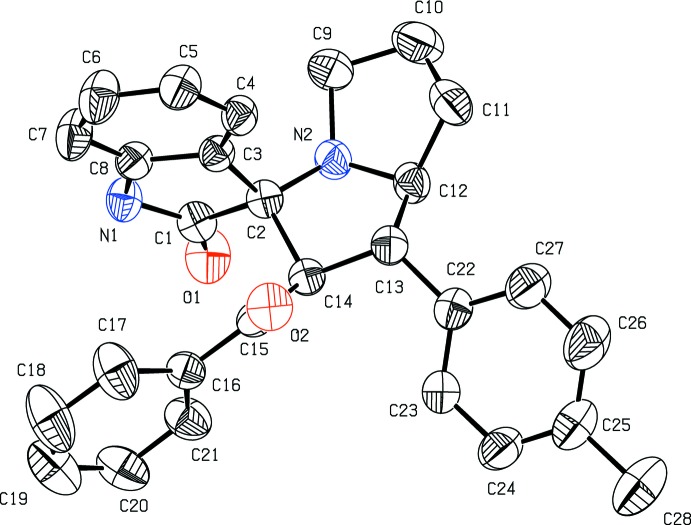
Displacement ellipsoid plot (50% probability level) of title compound (I), showing the atom-labelling scheme. H atoms have been omitted for clarity.

Mol­ecular diagrams of (I) and (II) are shown in Figs. 1 ▸ and 2 ▸, respectively. Both are di­spiro compounds in which the pyrrolizine and indole rings are spiro-fused, and they differ in the benzo­yl/methyl­phenyl and bromo­benzo­yl/chloro­phenyl substitutions. The Cremer & Pople puckering parameters of the two fused five-membered pyrrole rings of the pyrrolzine ring system in (I) *viz*. N2–C9–C10–C11–C12 and N2–C2–C14–C13–C12 are, respectively, *Q* = 0.362 (4) Å, φ = 264.4 (5)° indicating a twist about C10–C11, and *Q* = 0.408 (3) Å, φ=67.9 (4)° conforming to an envelope on C14. The corresponding values in (II), *Q* = 0.378 (3) Å, φ = 82.4 (4)° and *Q* = 0.423 (3) Å, φ = 251.4 (3)°, may differ slightly from those in (I) but they do not show a significant difference in the modes of puckering. The total puckering amplitude *Q* of the fused eight-membered pyrrolizine and the nine-membered indolone ring systems are respectively, 0.727 (3) and 0.129 (3) Å in (I) and 0.724 (2) and 0.065 (2) Å in (II), indicating that the atoms of the nine-membered indole ring system are nearly coplanar. In addition, the indole atom O1 remains coplanar with the rest of the atoms in both structures.

In both compounds, the spiro-fused ring systems tend to be rigid by remaining nearly perpendicular to each other, whereas the remaining substituted rings appear to be more ‘compromising’ towards hydrogen-bonding requirements, irrespective of their intra- or inter­molecular nature. As an example, the free rotation of the benzoyl group in (II) allows the formation of an intra­molecular C—H⋯O hydrogen bond (Table 2 ▸, last entry) while the inter­action is absent in (I).

**Figure 3:**
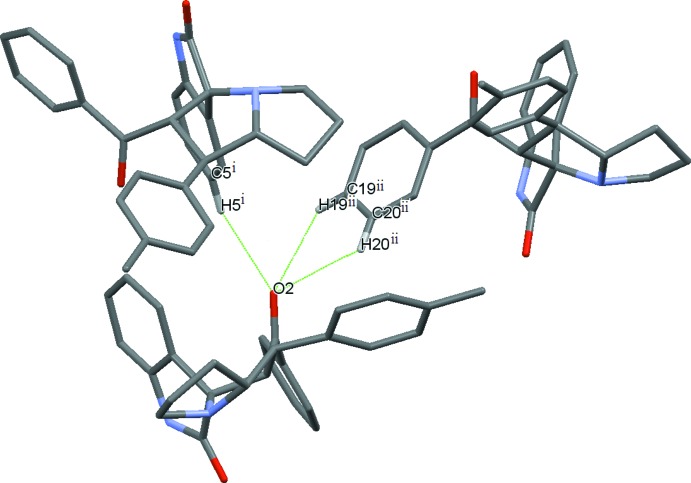
The three C—H⋯O bonds in (I) involving benzoyl O2 as acceptor (Table 1 ▸, top three entries) all from the same side of the plane.

**Figure 4:**
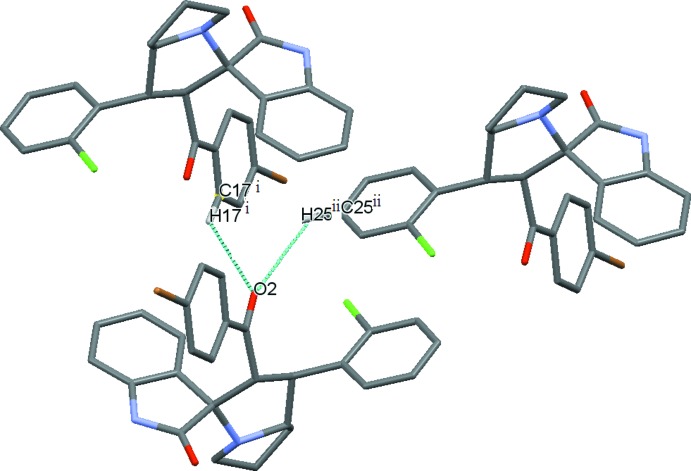
The two C—H⋯O bonds in (II) involving benzoyl O2 (Table 2 ▸, top two entries) on opposite sides of the benzoyl plane.

A significant difference between the two structures is observed in the deviation of benzoyl atom O2 from the least-squares plane of the C15–C21 atoms: 0.593 (4) in (I) and 0.131 (3) Å in (II). The larger deviation in (I) appears to be the result of the participation of O2 in three very weak (but cooperative) inter­molecular C—H⋯O hydrogen bonds, all three coming from the same side of the plane (Table 1 ▸, three topmost entries and Fig. 3 ▸). In the structure of (II), instead, only two (competitive) C—H⋯O bonds involving O2 occur, on opposite side of the plane (Table 2 ▸, two topmost entries and Fig. 4 ▸). These inter­molecular inter­action patterns exemplify a case where weak C—H⋯O hydrogen bonds can have noticeable effects on the mol­ecular conformation.

## Supra­molecular features   

**Figure 5:**
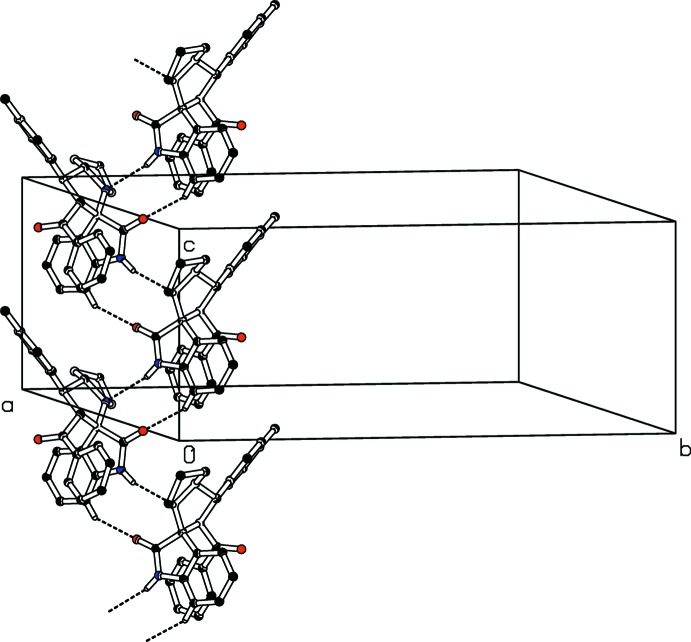
One-dimensional strings of mol­ecules of (I), along the *c* axis.

Even if the differences in the substituents produce differences in lattice types, space group, cell metrics, *etc*, these mol­ecular modifications do not seem to affect the type nor strength of the two relevant N—H⋯N and C—H⋯O inter­molecular hydrogen bonds defining the crystal structures (Tables 1 ▸ and 2 ▸), which can thus be considered as essential for the crystal structure layout. In particular, those bonds involving C7 and N1 link glide-related mol­ecules into similar one-dimensional strings along the shortest cell axis (Figs. 5 ▸ and 6 ▸). As already discussed, the other, relatively weaker, inter­molecular C—H⋯O hydrogen bonds involving the benzoyl atom O2 as acceptors have a profound effect on the mol­ecular conformation of the mol­ecules. Finally, a close O1⋯Br1(−*x* + 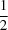, *y* − 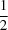, −*z* + 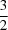) contact [*d*_O⋯Br_= 3.192 (2) Å] is present in structure (II), with no further significant Cl⋯Cl, Cl⋯Br, Br⋯Br or C—H⋯π or π–π inter­actions present in either crystal structure.

## Database survey   

A search of the Cambridge Structural Database (CSD, Version 5.53, update February 2014; Groom *et al.*, 2016 ▸) for organic non-polymeric single-crystal structures revealed 27 structures of which only two bear a close relationship to the title compound (POXZIL and POXZOR; Fokas *et al.*, 1998 ▸). There are no other direct analogues of the title compounds, either in coordinated or uncoordinated form. In POXZOR, the deviation of the benzoyl atom O2 from the plane containing the rest of the atoms of the group is about 0.465 Å, similar to the case in (I), but the quality of the H-atom treatment in POXZOR precluded any meaningful comparison.

## Synthesis and crystallization   

The synthesis of (I) involved a mixture of (*E*)-1-phenyl-3-(*p*-tol­yl)prop-2-en-1-one (0.4 mmol) [for the synthesis of (II), (*E*)-1-(4-bromo­phen­yl)-3-(2-chloro­phen­yl)prop-2-en-1-one (0.4 mmol)], isatin (0.4 mmol) and l-proline (0.4 mmol), which was dissolved in 5 ml of methanol, and 1 mol% of CMPTC (Chiral Multisite Phase Transfer Catalyst) was added and stirred at reflux temperature until the completion of reaction as indicated by TLC. After this step, the mixture was poured onto ice; the precipitate was filtered and recrystallized from ethanol solution, to get the pure product without column chromatography.

## Refinement   

Crystal data, data collection and structure refinement details are summarized in Table 3 ▸. In both (I) and (II), the carbon-bound H atoms were placed in calculated positions (C—H = 0.93–0.97 Å) and were included in the refinement in a riding-model approximation, with *U*_iso_(H) set at 1.2–1.5*U*_eq_(C). Compound (I) was refined as an inversion twin.

## Supplementary Material

Crystal structure: contains datablock(s) I, II, c1. DOI: 10.1107/S2056989016016741/bg2594sup1.cif

CCDC references: 1503430, 1503429

Additional supporting information: crystallographic information; 3D view; checkCIF report

## Figures and Tables

**Figure 2:**
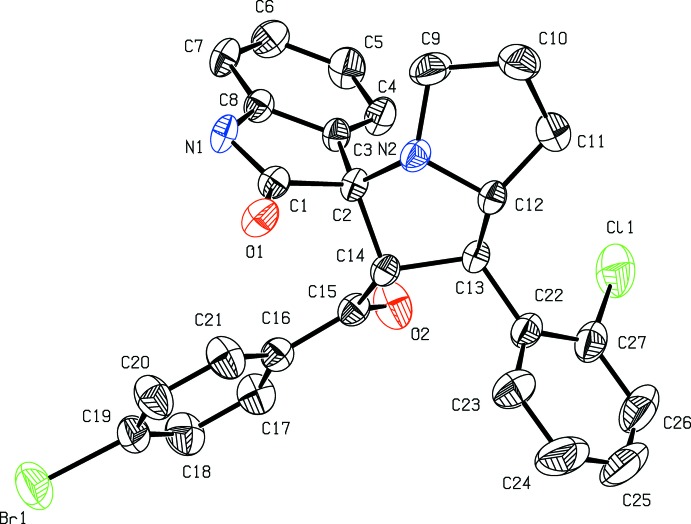
Displacement ellipsoid plot (50% probability level) of title compound (II), showing the atom-labelling scheme. H atoms have been omitted for clarity.

**Figure 6:**
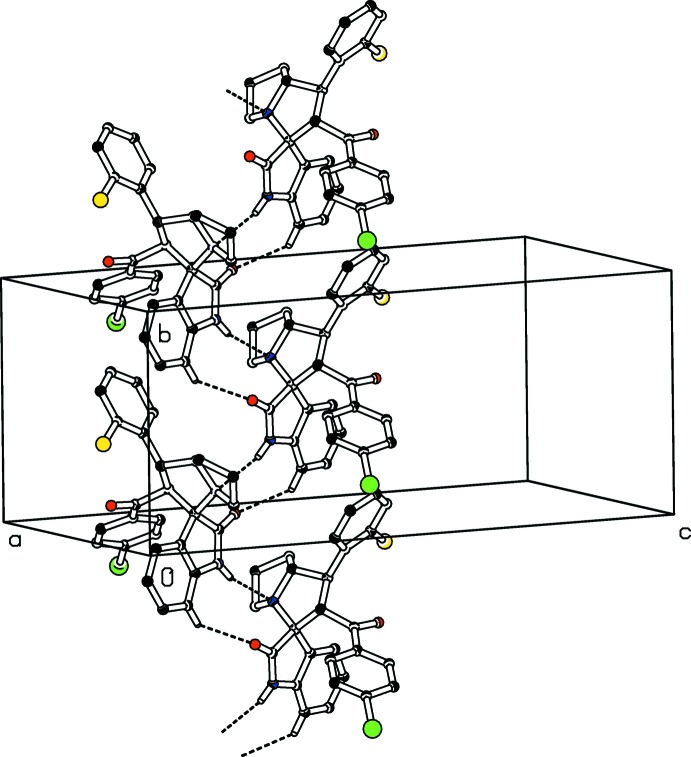
One-dimensional strings of mol­ecules of (II), along the *b* axis.

**Table 1:** Hydrogen-bond geometry (Å, °) for (I)

*D*—H⋯*A*	*D*—H	H⋯*A*	*D*⋯*A*	*D*—H⋯*A*
C5—H5⋯O2^i^	0.93	2.65	3.441 (4)	144
C19—H19⋯O2^ii^	0.93	2.70	3.301 (5)	123
C20—H20⋯O2^ii^	0.93	2.65	3.278 (4)	125
N1—H1⋯N2^iii^	0.86	2.40	3.232 (3)	163
C7—H7⋯O1^iii^	0.93	2.28	3.098 (4)	147

Symmetry codes: (i) 
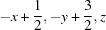
; (ii) 
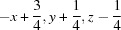
; (iii) 
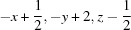
.

**Table 2:** Hydrogen-bond geometry (Å, °) for (II)

*D*—H⋯*A*	*D*—H	H⋯*A*	*D*⋯*A*	*D*—H⋯*A*
C17—H17⋯O2^i^	0.93	2.60	3.292 (3)	132
C25—H25⋯O2^ii^	0.93	2.61	3.327 (4)	134
N1—H1⋯N2^iii^	0.86	2.36	3.198 (3)	164
C7—H7⋯O1^iii^	0.93	2.47	3.235 (3)	139
C21—H21⋯O1	0.93	2.47	3.337 (3)	155

Symmetry codes: (i) 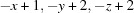; (ii) 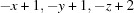; (iii) 
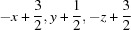
.

**Table 3:** Experimental details

	(I)	(II)
Crystal data		
Chemical formula	C_28_H_26_N_2_O_2_	C_27_H_22_BrClN_2_O_2_
*M*_r_	422.51	521.82
Crystal system, space group	Orthorhombic, *F* *d* *d*2	Monoclinic, *P*2_1_/*n*
Temperature (K)	295	295
*a*, *b*, *c* (Å)	36.030 (2), 24.2248 (16), 10.1301 (6)	10.7280 (4), 9.7793 (4), 22.6746 (9)
α, β, γ (°)	90, 90, 90	90, 98.972 (1), 90
*V* (Å^3^)	8841.7 (9)	2349.74 (16)
*Z*	16	4
Radiation type	Mo *K*α	Mo *K*α
μ (mm^−1^)	0.08	1.89
Crystal size (mm)	0.26 × 0.18 × 0.15	0.25 × 0.14 × 0.12

Data collection		
Diffractometer	Bruker SMART APEX CCD	Bruker SMARTAPEX CCD
Absorption correction	Multi-scan (*SADABS*; Bruker, 2009 ▸)	Multi-scan (*SADABS*; Bruker, 2009 ▸)
*T*_min_, *T*_max_	0.98, 0.99	0.89, 0.97
No. of measured, independent and observed [*I* > 2σ(*I*)] reflections	36903, 4649, 3325	22593, 4740, 3446
*R*_int_	0.108	0.029
(sin θ/λ)_max_ (Å^−1^)	0.636	0.622

Refinement		
*R*[*F* ^2^ > 2σ(*F* ^2^)], *wR*(*F* ^2^), *S*	0.041, 0.113, 0.98	0.039, 0.106, 1.03
No. of reflections	4649	4740
No. of parameters	291	298
No. of restraints	1	0
H-atom treatment	H-atom parameters constrained	H-atom parameters constrained
Δρ_max_, Δρ_min_ (e Å^−3^)	0.18, −0.14	0.64, −0.63
Absolute structure	Refined as a perfect inversion twin	–
Absolute structure parameter	0.5	–

Computer programs: *APEX2* and *SAINT* (Bruker, 2009 ▸), *SHELXS2013/1* (Sheldrick, 2008 ▸), *SHELXL2014/7* (Sheldrick, 2015 ▸), *PLUTON* (Spek, 2009 ▸), *Mercury* (Macrae *et al.*, 2006 ▸) and *publCIF* (Westrip, 2010 ▸).

## supplementary crystallographic information

### Crystal data

**Table:**

C_27_H_22_BrClN_2_O_2_	*F*(000) = 1064
*M**_r_* = 521.82	*D*_x_ = 1.475 Mg m^−^^3^
Monoclinic, *P*2_1_/*n*	Mo *K*α radiation, λ = 0.71073 Å
*a* = 10.7280 (4) Å	Cell parameters from 3446 reflections
*b* = 9.7793 (4) Å	θ = 2.0–26.0°
*c* = 22.6746 (9) Å	µ = 1.89 mm^−^^1^
β = 98.972 (1)°	*T* = 295 K
*V* = 2349.74 (16) Å^3^	Needle, colourless
*Z* = 4	0.25 × 0.14 × 0.12 mm

### Data collection

**Table:**

Bruker SMART APEX CCD diffractometer	3446 reflections with *I* > 2σ(*I*)
ω scans	*R*_int_ = 0.029
Absorption correction: multi-scan (SADABS; Bruker, 2009)	θ_max_ = 26.3°, θ_min_ = 2.0°
*T*_min_ = 0.89, *T*_max_ = 0.97	*h* = −13→13
22593 measured reflections	*k* = −12→12
4740 independent reflections	*l* = −28→25

### Refinement

**Table:**

Refinement on *F*^2^	0 restraints
Least-squares matrix: full	Hydrogen site location: inferred from neighbouring sites
*R*[*F*^2^ > 2σ(*F*^2^)] = 0.039	H-atom parameters constrained
*wR*(*F*^2^) = 0.106	*w* = 1/[σ^2^(*F*_o_^2^) + (0.0526*P*)^2^ + 0.9581*P*] where *P* = (*F*_o_^2^ + 2*F*_c_^2^)/3
*S* = 1.03	(Δ/σ)_max_ = 0.001
4740 reflections	Δρ_max_ = 0.64 e Å^−^^3^
298 parameters	Δρ_min_ = −0.63 e Å^−^^3^

### Special details

**Table:**

Geometry. All esds (except the esd in the dihedral angle between two l.s. planes) are estimated using the full covariance matrix. The cell esds are taken into account individually in the estimation of esds in distances, angles and torsion angles; correlations between esds in cell parameters are only used when they are defined by crystal symmetry. An approximate (isotropic) treatment of cell esds is used for estimating esds involving l.s. planes.

### Fractional atomic coordinates and isotropic or equivalent isotropic displacement parameters (Å^2^)

**Table:**

	*x*	*y*	*z*	*U*_iso_*/*U*_eq_	
Br1	0.07782 (3)	1.20198 (4)	0.80288 (2)	0.06695 (15)	
Cl1	0.77846 (8)	0.56854 (9)	1.02506 (3)	0.0637 (2)	
O1	0.63821 (17)	0.92263 (17)	0.73698 (7)	0.0378 (4)	
O2	0.57612 (18)	0.8610 (2)	0.95502 (8)	0.0471 (5)	
N1	0.72258 (19)	1.10554 (19)	0.79346 (9)	0.0312 (5)	
H1	0.7099	1.1686	0.7668	0.037*	
N2	0.82778 (19)	0.78758 (19)	0.82501 (9)	0.0305 (5)	
C1	0.6915 (2)	0.9728 (2)	0.78339 (10)	0.0283 (5)	
C2	0.7382 (2)	0.8914 (2)	0.84101 (10)	0.0266 (5)	
C3	0.7894 (2)	1.0043 (2)	0.88401 (10)	0.0272 (5)	
C4	0.8386 (2)	1.0042 (3)	0.94381 (11)	0.0364 (6)	
H4	0.8452	0.9230	0.9654	0.044*	
C5	0.8783 (3)	1.1264 (3)	0.97153 (12)	0.0419 (7)	
H5	0.9118	1.1271	1.0119	0.050*	
C6	0.8685 (3)	1.2472 (3)	0.93957 (12)	0.0393 (6)	
H6	0.8962	1.3282	0.9587	0.047*	
C7	0.8182 (3)	1.2497 (2)	0.87969 (11)	0.0350 (6)	
H7	0.8115	1.3309	0.8581	0.042*	
C8	0.7784 (2)	1.1277 (2)	0.85324 (10)	0.0278 (5)	
C9	0.9619 (3)	0.8258 (3)	0.82865 (15)	0.0489 (7)	
H9A	0.9835	0.8996	0.8570	0.059*	
H9B	0.9803	0.8543	0.7899	0.059*	
C10	1.0337 (3)	0.6969 (3)	0.84945 (15)	0.0517 (8)	
H10A	1.1173	0.7183	0.8704	0.062*	
H10B	1.0414	0.6371	0.8161	0.062*	
C11	0.9524 (2)	0.6323 (3)	0.89115 (13)	0.0458 (7)	
H11A	0.9675	0.6747	0.9303	0.055*	
H11B	0.9684	0.5349	0.8954	0.055*	
C12	0.8189 (2)	0.6601 (2)	0.85977 (10)	0.0283 (5)	
H12	0.7919	0.5849	0.8322	0.034*	
C13	0.7161 (2)	0.6880 (2)	0.89799 (10)	0.0265 (5)	
H13	0.7562	0.7282	0.9358	0.032*	
C14	0.6354 (2)	0.7997 (2)	0.86225 (10)	0.0262 (5)	
H14	0.5834	0.7582	0.8275	0.031*	
C15	0.5499 (2)	0.8689 (2)	0.90140 (11)	0.0298 (5)	
C16	0.4334 (2)	0.9432 (2)	0.87449 (11)	0.0313 (5)	
C17	0.3551 (2)	0.9935 (3)	0.91299 (12)	0.0406 (6)	
H17	0.3749	0.9766	0.9537	0.049*	
C18	0.2489 (3)	1.0680 (3)	0.89154 (13)	0.0470 (7)	
H18	0.1969	1.1008	0.9175	0.056*	
C19	0.2204 (2)	1.0934 (3)	0.83125 (13)	0.0430 (7)	
C20	0.2941 (3)	1.0426 (3)	0.79221 (13)	0.0510 (8)	
H20	0.2730	1.0587	0.7515	0.061*	
C21	0.4005 (3)	0.9672 (3)	0.81402 (12)	0.0431 (7)	
H21	0.4505	0.9321	0.7876	0.052*	
C22	0.6459 (2)	0.5618 (2)	0.91200 (11)	0.0344 (6)	
C23	0.5596 (3)	0.4974 (3)	0.86931 (15)	0.0517 (7)	
H23	0.5428	0.5342	0.8311	0.062*	
C24	0.4973 (3)	0.3791 (4)	0.88206 (19)	0.0724 (10)	
H24	0.4393	0.3376	0.8527	0.087*	
C25	0.5221 (4)	0.3242 (4)	0.9380 (2)	0.0744 (12)	
H25	0.4804	0.2450	0.9466	0.089*	
C26	0.6058 (3)	0.3830 (3)	0.98101 (17)	0.0630 (10)	
H26	0.6217	0.3450	1.0190	0.076*	
C27	0.6681 (3)	0.5007 (3)	0.96804 (13)	0.0434 (7)	

### Atomic displacement parameters (Å^2^)

**Table:**

	*U*^11^	*U*^22^	*U*^33^	*U*^12^	*U*^13^	*U*^23^
Br1	0.04738 (19)	0.0747 (3)	0.0810 (3)	0.02523 (17)	0.01691 (17)	0.02584 (19)
Cl1	0.0790 (6)	0.0694 (5)	0.0416 (4)	0.0123 (4)	0.0053 (4)	0.0191 (4)
O1	0.0523 (11)	0.0352 (10)	0.0250 (10)	−0.0092 (8)	0.0030 (8)	0.0001 (8)
O2	0.0537 (12)	0.0633 (13)	0.0253 (10)	0.0179 (10)	0.0087 (8)	0.0015 (9)
N1	0.0468 (12)	0.0211 (10)	0.0254 (11)	0.0009 (9)	0.0049 (9)	0.0051 (8)
N2	0.0343 (11)	0.0227 (10)	0.0366 (12)	0.0016 (9)	0.0127 (9)	0.0015 (9)
C1	0.0334 (12)	0.0286 (13)	0.0243 (12)	−0.0004 (10)	0.0087 (10)	0.0003 (10)
C2	0.0339 (12)	0.0206 (12)	0.0258 (12)	0.0011 (10)	0.0061 (10)	0.0017 (9)
C3	0.0321 (12)	0.0211 (12)	0.0280 (12)	0.0025 (10)	0.0034 (10)	−0.0002 (10)
C4	0.0485 (15)	0.0274 (13)	0.0308 (14)	−0.0036 (12)	−0.0015 (11)	0.0027 (11)
C5	0.0513 (16)	0.0397 (16)	0.0313 (15)	−0.0010 (13)	−0.0039 (12)	−0.0037 (12)
C6	0.0477 (15)	0.0273 (13)	0.0426 (16)	−0.0048 (12)	0.0061 (12)	−0.0099 (12)
C7	0.0486 (15)	0.0203 (12)	0.0374 (15)	0.0016 (11)	0.0103 (12)	0.0016 (11)
C8	0.0333 (12)	0.0228 (12)	0.0285 (13)	0.0020 (10)	0.0086 (10)	−0.0019 (10)
C9	0.0403 (15)	0.0387 (16)	0.073 (2)	−0.0053 (12)	0.0263 (15)	0.0014 (14)
C10	0.0353 (14)	0.0495 (18)	0.071 (2)	0.0010 (13)	0.0122 (14)	−0.0018 (15)
C11	0.0386 (15)	0.0458 (17)	0.0521 (18)	0.0064 (13)	0.0042 (13)	0.0085 (14)
C12	0.0351 (13)	0.0215 (12)	0.0282 (13)	0.0005 (10)	0.0046 (10)	0.0003 (10)
C13	0.0326 (12)	0.0208 (12)	0.0263 (12)	0.0007 (9)	0.0050 (10)	0.0017 (9)
C14	0.0314 (12)	0.0232 (12)	0.0241 (12)	−0.0005 (10)	0.0051 (9)	−0.0003 (9)
C15	0.0350 (13)	0.0255 (13)	0.0300 (14)	−0.0014 (10)	0.0086 (10)	−0.0012 (10)
C16	0.0344 (13)	0.0286 (13)	0.0316 (14)	0.0005 (10)	0.0072 (10)	−0.0026 (10)
C17	0.0447 (15)	0.0470 (16)	0.0315 (14)	0.0062 (13)	0.0106 (11)	−0.0032 (12)
C18	0.0455 (16)	0.0503 (18)	0.0487 (18)	0.0137 (14)	0.0187 (13)	−0.0024 (14)
C19	0.0355 (14)	0.0411 (16)	0.0534 (18)	0.0087 (12)	0.0104 (12)	0.0103 (13)
C20	0.0442 (16)	0.071 (2)	0.0386 (16)	0.0147 (15)	0.0092 (13)	0.0115 (15)
C21	0.0421 (15)	0.0562 (18)	0.0330 (15)	0.0135 (13)	0.0120 (12)	−0.0020 (13)
C22	0.0391 (14)	0.0252 (13)	0.0417 (15)	0.0021 (11)	0.0147 (12)	0.0036 (11)
C23	0.0572 (18)	0.0378 (16)	0.0604 (19)	−0.0119 (14)	0.0100 (15)	−0.0032 (14)
C24	0.068 (2)	0.052 (2)	0.099 (3)	−0.0265 (18)	0.017 (2)	−0.011 (2)
C25	0.082 (3)	0.0393 (19)	0.113 (3)	−0.0122 (18)	0.050 (3)	0.013 (2)
C26	0.079 (2)	0.0428 (19)	0.077 (2)	0.0060 (18)	0.043 (2)	0.0208 (17)
C27	0.0505 (16)	0.0317 (14)	0.0530 (18)	0.0096 (13)	0.0237 (13)	0.0100 (13)

### Geometric parameters (Å, º)

**Table:**

Br1—C19	1.891 (3)	C11—H11A	0.9700
Cl1—C27	1.743 (3)	C11—H11B	0.9700
O1—C1	1.220 (3)	C12—C13	1.530 (3)
O2—C15	1.207 (3)	C12—H12	0.9800
N1—C1	1.351 (3)	C13—C22	1.505 (3)
N1—C8	1.411 (3)	C13—C14	1.542 (3)
N1—H1	0.8600	C13—H13	0.9800
N2—C9	1.476 (3)	C14—C15	1.530 (3)
N2—C2	1.482 (3)	C14—H14	0.9800
N2—C12	1.486 (3)	C15—C16	1.492 (3)
C1—C2	1.545 (3)	C16—C21	1.382 (3)
C2—C3	1.518 (3)	C16—C17	1.392 (3)
C2—C14	1.555 (3)	C17—C18	1.375 (4)
C3—C4	1.376 (3)	C17—H17	0.9300
C3—C8	1.390 (3)	C18—C19	1.376 (4)
C4—C5	1.386 (4)	C18—H18	0.9300
C4—H4	0.9300	C19—C20	1.369 (4)
C5—C6	1.381 (4)	C20—C21	1.383 (4)
C5—H5	0.9300	C20—H20	0.9300
C6—C7	1.381 (4)	C21—H21	0.9300
C6—H6	0.9300	C22—C23	1.383 (4)
C7—C8	1.373 (3)	C22—C27	1.391 (4)
C7—H7	0.9300	C23—C24	1.388 (4)
C9—C10	1.514 (4)	C23—H23	0.9300
C9—H9A	0.9700	C24—C25	1.366 (6)
C9—H9B	0.9700	C24—H24	0.9300
C10—C11	1.520 (4)	C25—C26	1.347 (5)
C10—H10A	0.9700	C25—H25	0.9300
C10—H10B	0.9700	C26—C27	1.385 (4)
C11—C12	1.521 (3)	C26—H26	0.9300
			
C1—N1—C8	111.52 (19)	N2—C12—H12	109.1
C1—N1—H1	124.2	C11—C12—H12	109.1
C8—N1—H1	124.2	C13—C12—H12	109.1
C9—N2—C2	118.47 (19)	C22—C13—C12	113.70 (19)
C9—N2—C12	108.97 (19)	C22—C13—C14	115.66 (19)
C2—N2—C12	110.33 (17)	C12—C13—C14	102.94 (18)
O1—C1—N1	127.1 (2)	C22—C13—H13	108.1
O1—C1—C2	124.7 (2)	C12—C13—H13	108.1
N1—C1—C2	108.25 (19)	C14—C13—H13	108.1
N2—C2—C3	118.18 (19)	C15—C14—C13	110.26 (19)
N2—C2—C1	106.45 (18)	C15—C14—C2	116.27 (18)
C3—C2—C1	101.86 (18)	C13—C14—C2	101.78 (18)
N2—C2—C14	101.48 (17)	C15—C14—H14	109.4
C3—C2—C14	115.06 (19)	C13—C14—H14	109.4
C1—C2—C14	114.00 (19)	C2—C14—H14	109.4
C4—C3—C8	119.0 (2)	O2—C15—C16	119.5 (2)
C4—C3—C2	132.6 (2)	O2—C15—C14	119.3 (2)
C8—C3—C2	108.38 (19)	C16—C15—C14	121.2 (2)
C3—C4—C5	119.3 (2)	C21—C16—C17	118.4 (2)
C3—C4—H4	120.3	C21—C16—C15	123.9 (2)
C5—C4—H4	120.3	C17—C16—C15	117.7 (2)
C6—C5—C4	120.4 (2)	C18—C17—C16	120.9 (3)
C6—C5—H5	119.8	C18—C17—H17	119.6
C4—C5—H5	119.8	C16—C17—H17	119.6
C5—C6—C7	121.2 (2)	C17—C18—C19	119.4 (2)
C5—C6—H6	119.4	C17—C18—H18	120.3
C7—C6—H6	119.4	C19—C18—H18	120.3
C8—C7—C6	117.5 (2)	C20—C19—C18	121.0 (2)
C8—C7—H7	121.3	C20—C19—Br1	120.1 (2)
C6—C7—H7	121.3	C18—C19—Br1	118.8 (2)
C7—C8—C3	122.6 (2)	C19—C20—C21	119.3 (3)
C7—C8—N1	127.6 (2)	C19—C20—H20	120.3
C3—C8—N1	109.8 (2)	C21—C20—H20	120.3
N2—C9—C10	104.5 (2)	C16—C21—C20	121.0 (2)
N2—C9—H9A	110.8	C16—C21—H21	119.5
C10—C9—H9A	110.8	C20—C21—H21	119.5
N2—C9—H9B	110.8	C23—C22—C27	116.4 (3)
C10—C9—H9B	110.8	C23—C22—C13	121.9 (2)
H9A—C9—H9B	108.9	C27—C22—C13	121.7 (2)
C9—C10—C11	103.1 (2)	C22—C23—C24	121.7 (3)
C9—C10—H10A	111.1	C22—C23—H23	119.2
C11—C10—H10A	111.1	C24—C23—H23	119.2
C9—C10—H10B	111.1	C25—C24—C23	119.4 (4)
C11—C10—H10B	111.1	C25—C24—H24	120.3
H10A—C10—H10B	109.1	C23—C24—H24	120.3
C10—C11—C12	103.0 (2)	C26—C25—C24	121.0 (3)
C10—C11—H11A	111.2	C26—C25—H25	119.5
C12—C11—H11A	111.2	C24—C25—H25	119.5
C10—C11—H11B	111.2	C25—C26—C27	119.4 (3)
C12—C11—H11B	111.2	C25—C26—H26	120.3
H11A—C11—H11B	109.1	C27—C26—H26	120.3
N2—C12—C11	105.35 (19)	C26—C27—C22	122.1 (3)
N2—C12—C13	105.19 (18)	C26—C27—Cl1	116.9 (2)
C11—C12—C13	118.5 (2)	C22—C27—Cl1	120.9 (2)
			
C8—N1—C1—O1	177.4 (2)	N2—C12—C13—C14	24.7 (2)
C8—N1—C1—C2	−4.1 (3)	C11—C12—C13—C14	142.1 (2)
C9—N2—C2—C3	−25.9 (3)	C22—C13—C14—C15	71.0 (2)
C12—N2—C2—C3	100.6 (2)	C12—C13—C14—C15	−164.34 (18)
C9—N2—C2—C1	87.7 (3)	C22—C13—C14—C2	−164.97 (19)
C12—N2—C2—C1	−145.73 (19)	C12—C13—C14—C2	−40.4 (2)
C9—N2—C2—C14	−152.7 (2)	N2—C2—C14—C15	160.34 (19)
C12—N2—C2—C14	−26.2 (2)	C3—C2—C14—C15	31.5 (3)
O1—C1—C2—N2	58.4 (3)	C1—C2—C14—C15	−85.7 (2)
N1—C1—C2—N2	−120.2 (2)	N2—C2—C14—C13	40.5 (2)
O1—C1—C2—C3	−177.2 (2)	C3—C2—C14—C13	−88.3 (2)
N1—C1—C2—C3	4.2 (2)	C1—C2—C14—C13	154.52 (19)
O1—C1—C2—C14	−52.7 (3)	C13—C14—C15—O2	20.2 (3)
N1—C1—C2—C14	128.7 (2)	C2—C14—C15—O2	−94.9 (3)
N2—C2—C3—C4	−68.0 (3)	C13—C14—C15—C16	−158.4 (2)
C1—C2—C3—C4	175.8 (3)	C2—C14—C15—C16	86.4 (3)
C14—C2—C3—C4	52.0 (3)	O2—C15—C16—C21	174.7 (3)
N2—C2—C3—C8	113.3 (2)	C14—C15—C16—C21	−6.7 (4)
C1—C2—C3—C8	−2.9 (2)	O2—C15—C16—C17	−3.9 (4)
C14—C2—C3—C8	−126.7 (2)	C14—C15—C16—C17	174.7 (2)
C8—C3—C4—C5	−1.3 (4)	C21—C16—C17—C18	−1.4 (4)
C2—C3—C4—C5	−179.9 (2)	C15—C16—C17—C18	177.3 (2)
C3—C4—C5—C6	0.1 (4)	C16—C17—C18—C19	−0.3 (4)
C4—C5—C6—C7	0.5 (4)	C17—C18—C19—C20	1.8 (5)
C5—C6—C7—C8	0.0 (4)	C17—C18—C19—Br1	−177.5 (2)
C6—C7—C8—C3	−1.3 (4)	C18—C19—C20—C21	−1.4 (5)
C6—C7—C8—N1	178.9 (2)	Br1—C19—C20—C21	177.8 (2)
C4—C3—C8—C7	1.9 (4)	C17—C16—C21—C20	1.7 (4)
C2—C3—C8—C7	−179.2 (2)	C15—C16—C21—C20	−176.8 (3)
C4—C3—C8—N1	−178.2 (2)	C19—C20—C21—C16	−0.4 (5)
C2—C3—C8—N1	0.7 (3)	C12—C13—C22—C23	−73.0 (3)
C1—N1—C8—C7	−177.9 (2)	C14—C13—C22—C23	45.8 (3)
C1—N1—C8—C3	2.2 (3)	C12—C13—C22—C27	104.4 (3)
C2—N2—C9—C10	144.5 (2)	C14—C13—C22—C27	−136.8 (2)
C12—N2—C9—C10	17.3 (3)	C27—C22—C23—C24	0.9 (4)
N2—C9—C10—C11	−34.6 (3)	C13—C22—C23—C24	178.4 (3)
C9—C10—C11—C12	38.6 (3)	C22—C23—C24—C25	−0.3 (5)
C9—N2—C12—C11	6.9 (3)	C23—C24—C25—C26	0.0 (6)
C2—N2—C12—C11	−124.8 (2)	C24—C25—C26—C27	−0.3 (5)
C9—N2—C12—C13	132.8 (2)	C25—C26—C27—C22	0.9 (5)
C2—N2—C12—C13	1.2 (2)	C25—C26—C27—Cl1	−178.2 (3)
C10—C11—C12—N2	−28.2 (3)	C23—C22—C27—C26	−1.2 (4)
C10—C11—C12—C13	−145.4 (2)	C13—C22—C27—C26	−178.7 (2)
N2—C12—C13—C22	150.6 (2)	C23—C22—C27—Cl1	177.9 (2)
C11—C12—C13—C22	−92.0 (3)	C13—C22—C27—Cl1	0.4 (3)

### Hydrogen-bond geometry (Å, º)

**Table:**

*D*—H···*A*	*D*—H	H···*A*	*D*···*A*	*D*—H···*A*
C17—H17···O2^i^	0.93	2.60	3.292 (3)	132
C25—H25···O2^ii^	0.93	2.61	3.327 (4)	134
N1—H1···N2^iii^	0.86	2.36	3.198 (3)	164
C7—H7···O1^iii^	0.93	2.47	3.235 (3)	139
C21—H21···O1	0.93	2.47	3.337 (3)	155

Symmetry codes: (i) −*x*+1, −*y*+2, −*z*+2; (ii) −*x*+1, −*y*+1, −*z*+2; (iii) −*x*+3/2, *y*+1/2, −*z*+3/2.
